# Evolutionary Games of Multiplayer Cooperation on Graphs

**DOI:** 10.1371/journal.pcbi.1005059

**Published:** 2016-08-11

**Authors:** Jorge Peña, Bin Wu, Jordi Arranz, Arne Traulsen

**Affiliations:** 1 Department of Evolutionary Theory, Max Planck Institute for Evolutionary Biology, Plön, Germany; 2 School of Sciences, Beijing University of Posts and Telecommunications, Beijing, China; University of California, Irvine, UNITED STATES

## Abstract

There has been much interest in studying evolutionary games in structured populations, often modeled as graphs. However, most analytical results so far have only been obtained for two-player or linear games, while the study of more complex multiplayer games has been usually tackled by computer simulations. Here we investigate evolutionary multiplayer games on graphs updated with a Moran death-Birth process. For cycles, we obtain an exact analytical condition for cooperation to be favored by natural selection, given in terms of the payoffs of the game and a set of structure coefficients. For regular graphs of degree three and larger, we estimate this condition using a combination of pair approximation and diffusion approximation. For a large class of cooperation games, our approximations suggest that graph-structured populations are stronger promoters of cooperation than populations lacking spatial structure. Computer simulations validate our analytical approximations for random regular graphs and cycles, but show systematic differences for graphs with many loops such as lattices. In particular, our simulation results show that these kinds of graphs can even lead to more stringent conditions for the evolution of cooperation than well-mixed populations. Overall, we provide evidence suggesting that the complexity arising from many-player interactions and spatial structure can be captured by pair approximation in the case of random graphs, but that it need to be handled with care for graphs with high clustering.

## Author Summary

Cooperation can be defined as the act of providing fitness benefits to other individuals, often at a personal cost. When interactions occur mainly with neighbors, assortment of strategies can favor cooperation but local competition can undermine it. Previous research has shown that a single coefficient can capture this trade-off when cooperative interactions take place between two players. More complicated, but also more realistic, models of cooperative interactions involving multiple players instead require several such coefficients, making it difficult to assess the effects of population structure. Here, we obtain analytical approximations for the coefficients of multiplayer games in graph-structured populations. Computer simulations show that, for particular instances of multiplayer games, these approximate coefficients predict the condition for cooperation to be promoted in random graphs well, but fail to do so in graphs with more structure, such as lattices. Our work extends and generalizes established results on the evolution of cooperation on graphs, but also highlights the importance of explicitly taking into account higher-order statistical associations in order to assess the evolutionary dynamics of cooperation in spatially structured populations.

## Introduction

Graphs are a natural starting point to assess the role of population structure in the evolution of cooperation. Vertices of the graph represent individuals, while links (edges) define interaction and dispersal neighborhoods. Classical models of population structure, such as island models [1, 2] and lattices [3, 4], often developed before the current interest in complex networks [5, 6], can all be understood as particular instances of graphs [7, 8]. More recently, the popularity of network theory has fueled a renewed interest in evolutionary dynamics on graphs, especially in the context of social behaviors such as cooperation and altruism [7–21].

When selection is weak on two competing strategies, such that fitness differences represent only a small perturbation of a neutral evolutionary process, a surprisingly simple condition for one strategy to dominate the other, known as the “sigma rule”, holds for a large variety of graphs and other models of spatially structured populations [22]. Such a condition depends not only on the payoffs of the game describing the social interactions, but also on a number of “structure coefficients”. These coefficients are functions of demographic parameters of the spatial model and of its associated update protocol, but are independent of the payoffs. In the case of two-player games, the sigma rule depends on a single structure coefficient *σ*. The larger this *σ*, the greater the ability of spatial structure to promote the evolution of cooperation or to choose efficient equilibria in coordination games [22]. Partly for this reason, the calculation of structure coefficients for different models of population structure has attracted significant interest during the last years [8, 21–27].

Despite the theoretical and empirical importance of two-player games, many social interactions involve the collective action of more than two individuals. Examples range from bacteria producing extracellular compounds [28–31] to human social dilemmas [32–36]. In these situations, the evolution of cooperation is better modeled as a multiplayer game where individuals obtain their payoffs from interactions with more than two players [37–43]. An example of such multiplayer games is the volunteer’s dilemma, where individuals in a group must decide whether to volunteer (at a personal cost) or to ignore, knowing that volunteering from at least one individual is required for a public good to be provided [44–46]. Importantly, such a multiplayer interaction cannot be represented as a collection of pairwise games, because changes in payoff are nonlinear in the number of co-players choosing a particular action.

Multiplayer games such as the volunteer’s dilemma can also be embedded in graphs, assuming, for instance, that nodes represent both individuals playing games and games played by individuals [47–49]. Most previous studies on the effects of graph structure on multiplayer game dynamics have relied on computer simulations [49]. However, similar to the two-player case, some analytical progress can be made if selection is assumed to be weak. In the multiplayer case, the sigma rule depends no longer on one, but on up to *d* − 1 structure coefficients, where *d* is the number of players [50]. Although exact formulas for structure coefficients of multiplayer games can be obtained for relatively simple models such as cycles [51], analysis has proved elusive in more complex population structures, including regular graphs of arbitrary degree. Indeed, extending analytical results on evolutionary two-player games on graphs to more general multiplayer games is an open problem in evolutionary graph theory [52].

Here, we contribute to this body of work by deriving approximate analytical expressions for the structure coefficients of regular graphs updated with a Moran death-Birth model, and hence for the condition of one strategy to dominate another according to the sigma rule. The expressions we find for the structure coefficients suggest that regular graphs updated with a Moran death-Birth model lead to less stringent conditions for the evolution of cooperation than those characteristic of well-mixed populations. Computer simulations suggest that our approximations are good for random regular graphs, but that they systematically overestimate the condition for the evolution of cooperation in graphs with more loops and higher clustering such as rings and lattices. In these cases, cooperation can be no longer promoted, but even be hindered, with respect to the baseline case of a population lacking spatial structure.

## Methods

We consider stochastic evolutionary dynamics on a graph-structured population of size *N*. Each individual is located at the vertex of a regular graph of degree *k*. Individuals obtain a payoff by interacting with their *k* neighbors in a single *d*-person symmetric game (i.e., *d* = *k*+1). If *j* co-players play *A*, a focal *A*-player obtains *a*_*j*_ whereas a focal *B*-player obtains *b*_*j*_, as indicated in Table 1.

**Table 1:** Payoffs to *A*-players and *B*-players.

Opposing *A*-players	0	1	…	*j*	…	*d* − 1
payoff to *A*	*a*_0_	*a*_1_	…	*a*_*j*_	…	*a*_*d*−1_
payoff to *B*	*b*_0_	*b*_1_	…	*b*_*j*_	…	*b*_*d*−1_

We model the stochastic evolutionary dynamics as a Markov process on a finite space state. More specifically, we consider a Moran death-Birth process [12, 14, 53] according to which, at each time step: (i) a random individual is chosen to die, and (ii) its neighbors compete to place a copy of themselves in the new empty site with probability proportional to 1 − *w* + *w* × *payoff*, where the parameter *w* measures the intensity of selection. Without mutation, such a Markov process has two absorbing states: one where all vertices are occupied by *A*-players and one where all vertices are occupied by *B*-players. Let us denote by *ρ*_*A*_ the fixation probability of a single *A*-player in a population of *B*-players, and by *ρ*_*B*_ the fixation probability of a single *B*-player in a population of *A*-players. We take the comparison of fixation probabilities, i.e.

$$\rho_{A}>\rho_{B},$$ (1)

as a measure of evolutionary success [54] and say that *A* is favored over *B* if condition (1) holds.

Under weak selection (i.e., *w* ≪ 1) the condition for *A* to be favored over *B* holds if the sigma rule for multiplayer games [50] is satisfied, i.e., if

$$\sum_{j=0}^{d-1}\sigma_{j}f_{j}>0,$$ (2)

where *σ*_0_, …, *σ*_*d*−1_ are the *d* structure coefficients (constants that depend on the population structure and on the update dynamics), and

$$f_{j}=a_{j}-b_{d-1-j},\;\;j=0,1,\ldots ,d-1,$$ (3)

are differences between payoffs, which we will refer to in the following as the “gains from flipping”. The gains from flipping capture the change in payoff experienced by a focal individual playing *B* in a group where *j* co-players play *A* when all players simultaneously switch strategies (so that *A*-players become *B*-players and *B*-players become *A*-players). It turns out that the payoffs of the game only enter into condition (1) via the gains from flipping Eq (3), as the structure coefficients are themselves independent of *a*_*j*_ and *b*_*j*_.

Structure coefficients are uniquely determined up to a constant factor. Setting one of these coefficients to one thus gives a single structure coefficient for *d* = 2 [22]. For *d* > 2, and in the usual case where structure coefficients are nonnegative, we can impose $\sum_{j=0}^{d-1}\sigma_{j}=1$ without affecting the selection condition (2). For our purposes, this normalization turns out to be more useful than setting one coefficient to one, as it allows us to rewrite the sigma rule Eq (2) as

$$\sum_{j=0}^{d-1}\varsigma_{j}f_{j}=E\left[f(J)\right]>0,$$ (4)

where *f*(*j*) ≡ *f*_*j*_, and *J* is the random variable with probability distribution prescribed by the “normalized structure coefficients” $\varsigma_{j}=\sigma_{j}/\sum_{i=0}^{d-1}\sigma_{i}$. In light of condition (4), the sigma rule can be interpreted as stating that strategy *A* is favored over *B* if the expected gains from flipping are greater than zero when the number of co-players *J* is distributed according to the normalized structure coefficients. From this perspective, different models of population structure lead to different normalized structured coefficients and hence to different expected gains from flipping, which in turn imply different conditions for strategy *A* to be favored over *B* in a given multiplayer game [51]. For instance, a well-mixed population with random group formation updated with either a Moran or a Wright-Fisher process leads to normalized structure coefficients given by [39, 40]:

$$\varsigma_{j}^{W}=\left\{\begin{array}{cc} \frac{N}{d(N-1)} & \text{if}\;0\leq j\leq d-2\\ \frac{N-d}{d(N-1)} & \text{if}\;j=d-1 \end{array}\right..$$ (5)

A normalized sigma rule such as the one given by Eq (4) holds for many spatial models and associated updating protocols [50, 51]. Here, we focus on the case of regular graphs updated with a Moran death-Birth process. We provide exact expressions for the case of cycles for which *k* = 2. For *k* ≥ 3, we bypass the difficulties of an exact calculation by using a combination of pair approximation [55, 56] and diffusion approximation [14]. Our approach implicitly assumes that graphs are equivalent to Bethe lattices (or Cayley trees) with a very large number of vertices (*N* ≫ *k*). In addition, weak selection intensities (*wk* ≪ 1) are also required for an implicit argument of separation of timescales to hold. In order to assess the validity of our analytical approximations, we implemented a computational model of a Moran death-Birth process in three different types of regular graphs (rings, random graphs, and lattices) with different degrees and estimated numerically the fixation probabilities *ρ*_*A*_ and *ρ*_*B*_ as the proportion of realizations where the mutant succeeded in invading the wild-type.

## Results

### Exact structure coefficients and sigma rule for cycles

Going beyond the complete graph representing a well-mixed population, the simplest case of a regular graph is the cycle, for which *k* = 2 (and consequently *d* = 3). In this case, we find the following exact expressions for the structure coefficients (S1 Text, Section 1):

$$\varsigma_{0}^{G}=\frac{1}{2(N-2)},\;\varsigma_{1}^{G}=\frac{1}{2},\;\varsigma_{2}^{G}=\frac{N-3}{2(N-2)}.$$ (6)

For large *N*, the structure coefficients reduce to $\varsigma_{0}^{G}=0$, $\varsigma_{1}^{G}=\varsigma_{2}^{G}=1/2$ and the sigma rule Eq (4) simplifies to

$$a_{1}+a_{2}>b_{1}+b_{0}.$$ (7)

**Fig 1:**
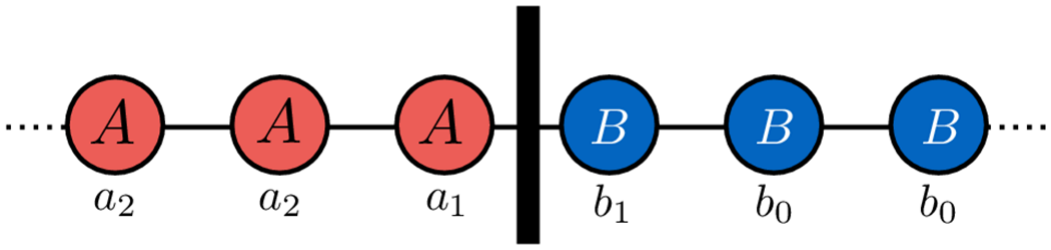
Payoffs at the boundary of two clusters in the cycle. Under weak selection, the cluster of *A*-players expands if the sigma rule *a*_1_+*a*_2_ > *b*_1_+*b*_0_ holds. As a player is never paired with two players of the opposite strategy, neither *a*_0_ nor *b*_2_ enter into this expression. This provides an intuition behind our analytical results in the simple case when the graph is a cycle.

This is also the condition for the boundary between a cluster of *A*-players and a cluster of *B*-players to move in favor of *A*-players for weak selection [57] (Fig 1). Condition (7) implies that *A* can be favored over *B* even if *A* is strictly dominated by *B* (i.e., *a*_*j*_ < *b*_*j*_ for all *j*) as long as the payoff for mutual cooperation *a*_2_ is large enough so that *a*_2_ > *b*_0_+(*b*_1_ − *a*_1_); a necessary condition for this inequality to hold is that *A* strictly Pareto dominates *B* (i.e., *a*_2_ > *b*_0_). Such a result is impossible in well-mixed populations, where the structure coefficients Eq (5) prevent strictly dominated strategies from being favored by selection. Condition (7) provides a simple example of how spatial structure can affect evolutionary game dynamics and ultimately favor the evolution of cooperation and altruism.

### Approximate structure coefficients and sigma rule for regular graphs of degree *k* ≥ 3

For regular graphs of degree *k* ≥ 3, we find that the structure coefficients can be approximated by (S1 Text, Section 2)

$$\varsigma_{j}^{G}=\frac{{\left(k-2\right)}^{k-1-j}}{\left(k+2\right)\left(k+1\right)k^{2}}\sum_{\ell =0}^{k-1}\left(k-\ell \right)\left\{\left[k^{2}-\left(k-2\right)\ell \right]\upsilon_{\ell ,j,k}+\left[2k+(k-2)\ell \right]\tau_{\ell ,j,k}\right\},$$ (8)

where

$$\upsilon_{l,j,k}=\left(\begin{array}{c} k-1-l\\ k-1-j \end{array}\right)\frac{1}{{\left(k-1\right)}^{k-1-l}}+\left(\begin{array}{c} l\\ k-j \end{array}\right)\frac{k-2}{{\left(k-1\right)}^{l}},$$ (9)

and

$$\tau_{l,j,k}=\left(\begin{array}{c} k-1-l\\ k-j \end{array}\right)\frac{k-2}{{\left(k-1\right)}^{k-1-l}}+\left(\begin{array}{c} l\\ k-1-j \end{array}\right)\frac{1}{{\left(k-1\right)}^{l}}.$$ (10)

These expressions are nontrivial functions of the degree of the graph *k* and thus difficult to interpret. For instance, for *k* = 3, we obtain $\varsigma^{G}=(\frac{7}{144},\frac{31}{144},\frac{61}{144},\frac{45}{144})$.

### Promotion of multiplayer cooperation

The previous results hold for any symmetric multiplayer game with two strategies. To investigate the evolution of multiplayer cooperation, let us label strategy *A* as “cooperate”, strategy *B* as “defect”, and assume that, irrespective of the focal player’s strategy, the payoff of a focal player increases with the number of co-players playing *A*, i.e.,

$$a_{j+1}\geq a_{j}\;\text{and}\;b_{j+1}\geq b_{j}\;\text{for}\;\text{all}\;j.$$ (11)

This restriction on the payoffs is characteristic of “cooperation games” [51] in which playing *A* is beneficial to the group but might be costly to the individual. Well-known multiplayer games belonging to this large class of games include different instances of volunteer’s dilemmas [44, 46], snowdrift games [58], stag hunts [59], and many other instances of public, club, and charity goods games [43].

**Fig 2:**
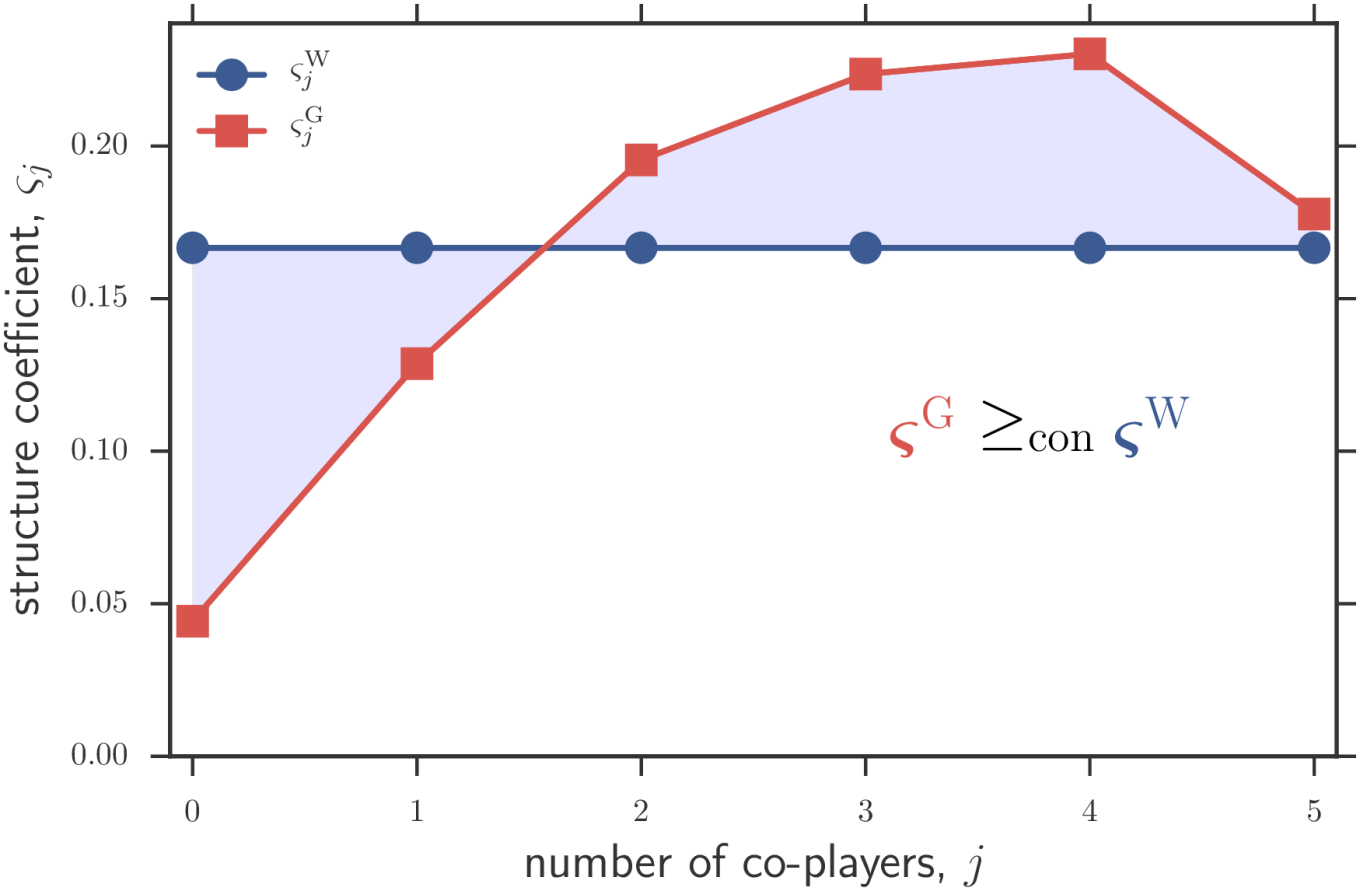
Structure coefficients and containment order of cooperation. Approximated (normalized) structure coefficients ς_*j*_ for large regular graphs of degree *k* = 5 updated with a Moran death-Birth process ($\varsigma_{j}^{G}$) and large well-mixed populations where groups of *d* = 6 players are randomly matched to play a game ($\varsigma_{j}^{W}$). Since ς^G^ − ς^W^ has one sign crossing from − to +, the graph is greater in the containment order than the well-mixed population (denoted by $\varsigma^{G}\geq_{\text{con}}\varsigma^{W}$). Consequently, if the sigma rule holds for a well-mixed population with coefficients ς^W^, then it also holds for a graph-structured population with coefficients ς^G^, for any cooperation game.

We are interested in establishing whether graph-structured populations systematically lead to structure coefficients that make it easier to satisfy the normalized sigma rule Eq (4) than well-mixed populations (the baseline case scenario of a population with no spatial structure) for any cooperation game satisfying condition (11). In other words, we ask whether a graph is a stronger promoter of cooperation than a well-mixed population. Technically, this is equivalent to asking whether the set of games for which cooperation is favored under a graph contains the set of games for which cooperation is favored under a well-mixed population, i.e., whether a graph is greater than a well-mixed population in the “containment order” [51]. A simple sufficient condition for this is that the difference in normalized structure coefficients, ς^G^ − ς^W^, has exactly one sign change from − to + [51]. This can be verified for any *N* > 3 in the case of cycles (*k* = 2) by inspection of eqs (5) and (6). For large regular graphs of degree *k* ≥ 3 and hence multiplayer games with *d* ≥ 4 players, we checked the condition numerically by comparing eqs (5) and (8) for *k* = 3, …, 100. We find that ς^G^ − ς^W^ always has a single sign change from − to + and hence that, in the limit of validity of our approximations, regular graphs promote more cooperation than well-mixed populations for all games fulfilling Eq (11) (Fig 2). In the following, we explore in more detail the sigma rule for particular examples of multiplayer games.

### Examples

#### Collections of two-player games

As a consistency check, let us consider the case where individuals play two-player games with their *k* neighbors and collect the payoffs of the different interactions. The two-player game is given by the payoff matrix

$$\begin{array}{l} \begin{array}{ccc} & A & B \end{array}\\ \begin{array}{c} A\\ B \end{array}\left(\begin{array}{cc} \alpha & \beta \\ \gamma & \delta \end{array}\right) \end{array}.$$ (12)

The payoffs for the resulting multiplayer game, which are just the sum of payoffs of the pairwise games, are then given by *a*_*j*_ = *jα* + (*k* − *j*)*β* and *b*_*j*_ = *jγ* + (*k* − *j*)*δ*. The sigma rule Eq (4) can hence be written as

$$k\left(\beta -\gamma \right)+\left(\alpha -\beta +\gamma -\delta \right)\sum_{j=0}^{k}\varsigma_{j}^{G}j>0.$$ (13)

We can show that (S1 Text, Section 2.9)

$$\sum_{j=0}^{k}\varsigma_{j}^{G}j=E\left[J\right]=\frac{k+1}{2},$$ (14)

so that condition (13) is equivalent to

$$\left(k+1\right)\alpha +\left(k-1\right)\beta -\left(k-1\right)\gamma -\left(k+1\right)\delta >0,$$ (15)

i.e., the sigma rule previously established for pairwise games in regular graphs [cf. Eq (24) in the Supplementary Material of Ref. [14]]. For a pairwise donation game (for which α=B-C, β=-C, γ=B, *δ* = 0, where B and C are respectively the benefit and cost of donation) this reduces to the well-known B/C>k rule [7, 14, 16].

#### Linear games

Suppose now that *a*_*j*_ and *b*_*j*_ are both linear functions of *j*. We can thus write $a_{j}=-C+\left(B+D\right)j/k$, $b_{j}=Bj/k$ for some parameters B, C, and D. When B>C≥0, such a game can be interpreted in terms of a social dilemma as follows. Cooperators each pay a cost C in order to provide a benefit B/k to each of their co-players; defectors receive the benefits but pay no cost. In addition to the benefit B/k, cooperators also get an additional bonus D/k per other cooperator in the group. This bonus can be positive or negative.

For such linear games, and by making use of Eq (14), the sigma condition simplifies to 2B+D(k+1)>2Ck. When there is no bonus (D=0) the game is an additive prisoner’s dilemma [60] and we recover the condition B/C>k. In the limit of large *k*, the sigma condition becomes D>2C.

#### Volunteer’s dilemma

As an example of a nonlinear multiplayer game satisfying condition (11), consider the volunteer’s dilemma [44, 45]. In such a game, one cooperator can produce a public good of value B at a personal cost C; defectors pay no cost and provide no benefit. Payoffs are then given by $a_{j}=B-C$ for all *j*, *b*_0_ = 0, and $b_{j}=B$ for *j* > 0. The sigma rule, Eq (4), for the volunteer’s dilemma hence reduces to

$$B/C>1/\varsigma_{d-1}.$$ (16)

For the cycle, we thus find

$$B/C>\frac{2(N-2)}{N-3},$$ (17)

which in the limit of large *N* reduces to B/C>2. For large regular graphs of degree *k* ≥ 3, our approximations lead to

$$B/C>\frac{k(k+1)(k-2)}{{\left(k-1\right)}^{2}-{\left(k-1\right)}^{1-k}}.$$ (18)

These conditions contrast with that for a large well mixed population, which is given by B/C>k+1.

Suppose now that the cost of producing the public good is shared among cooperators [46]. Payoffs are then given by $a_{j}=B-C/\left(j+1\right)$, *b*_0_ = 0 and $b_{j}=B$ for *j* > 0. In this case the sigma rule simplifies to

$$B/C>\frac{1}{\varsigma_{d-1}}\sum_{j=0}^{d-1}\frac{\varsigma_{j}}{j+1}.$$ (19)

This leads to

$$B/C>\frac{5N-6}{6(N-3)}$$ (20)

in the case of a finite cycle of size *N* and B/C>5/6 for a large cycle. Contrastingly, in a well-mixed population,

$$B/C>\sum_{j=0}^{d-1}\frac{1}{j+1}.$$ (21)

### Computer simulations

**Fig 3:**
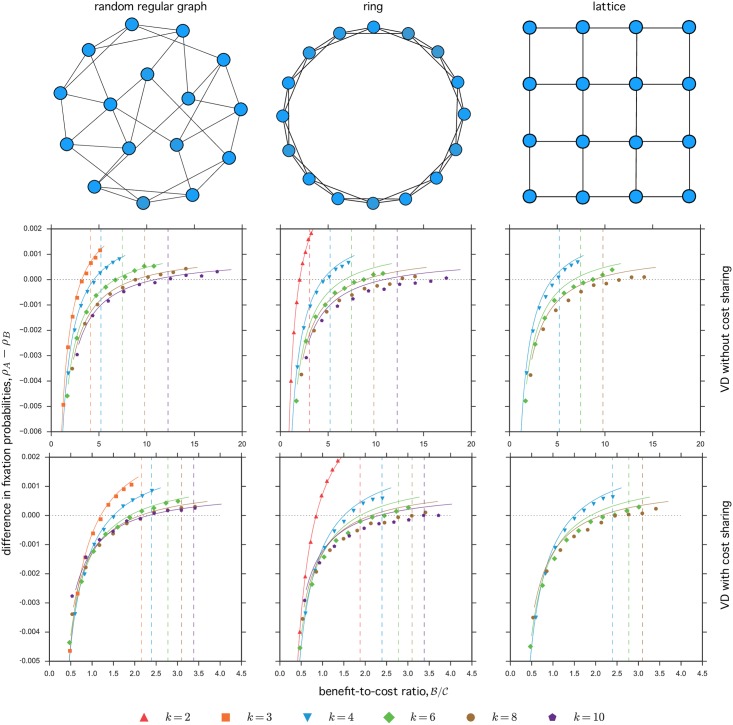
Simulations of evolutionary game dynamics on graphs (difference in fixation probabilities, *N* = 100). The first row shows the type of (regular) graph for the particular case of *k* = 4, i.e., each node has exactly four neighbors. The second and third rows show simulation results for the volunteer’s dilemma without cost-sharing and with cost-sharing, respectively. Simulation data in the first column correspond to random regular graphs, in the second column to rings, and in the third column to lattices. The fixation probability of cooperators, *ρ*_*A*_ (defectors, *ρ*_*B*_) was calculated as the fraction of runs where a single cooperator (defector) reached fixation out of 10^7^ runs. Symbols show the difference between such fixation probabilities, as a function of the benefit-to-cost ratio B/C, for different types and degrees of the graph. Lines indicate analytical predictions for the difference in fixation probabilities (left hand side of Eq (4) with normalized sigmas given by Eqs (6) or (8)). Dashed vertical lines the critical benefit-to-cost ratios B/C above which we have *ρ*_*A*_ > *ρ*_*B*_ for well-mixed populations (right hand side of Eqs (16) or (19) with normalized sigmas given by Eq (5)). Parameters: population size *N* = 100, intensity of selection *w* = 0.01, payoff cost C=1.

To assess the validity of our approximations, we compare our analytical results with explicit simulations of evolutionary dynamics on graphs (Fig 3, *N* = 100; S1 Fig, *N* = 500). We implemented three different kinds of regular graphs: (i) random regular graphs, (ii) rings (generalized cycles in which each node is connected to *k*/2 nodes to the left and *k*/2 nodes to the right), and (iii) lattices (a square lattice with von Neumann neighborhood with *k* = 4, a hexagonal lattice with *k* = 6, and a square lattice with Moore neighborhood and *k* = 8). Analytical predictions are in good agreement with simulation results in the case of cycles (i.e., rings with *k* = 2, for which our expressions are exact) and for all random regular graphs that we explored. Contrastingly, for rings with *k* ≥ 4 and lattices, our approximations tend to underestimate the critical benefit-to-cost ratio beyond which the fixation probability of cooperators is greater than that of defectors. In other words, our analytical results seem to provide necessary but not sufficient conditions for cooperation to be favored. Such discrepancies stem from the fact that our analysis assumes graphs with no loops such as Cayley trees; the error induced by our approximations is more evident when looking at the actual fixation probabilities (S2 Fig, *N* = 100, S3 Fig, *N* = 500) and not just at their difference. As all graphs with *k* > 2 we considered do contain loops, such mismatch is expected—in particular for rings and lattices, which are characterized by high clustering. Perhaps more importantly, our simulations suggest that the critical benefit-to-cost ratio for the volunteer’s dilemma without cost sharing in rings and lattices with *k* ≥ 6 is greater than the corresponding values for random graphs and well-mixed populations. This illustrates a case in which a graph-structured population updated with a death-Birth process leads to less favorable conditions for the evolution of cooperation than a well-mixed population.

## Discussion

We studied evolutionary multiplayer game dynamics on graphs, focusing on the case of a Moran death-Birth process on regular structures. First, we used a combination of pair approximation and diffusion approximation to provide analytical formulas for the structure coefficients of a regular graph, which together with the payoffs from the game determine when a strategy is more abundant than another in the limits of weak selection and weak mutation. Such a condition is valid for any symmetric multiplayer game, including the volunteer’s dilemma [44–46] and other multiplayer social dilemmas discussed in the recent literature [38, 41, 58, 59, 61]. The condition can be used to determine the specific conditions (in terms of the degree of the graph and the parameters of the game, such as payoff costs and benefits) under which cooperation will thrive. The structure coefficients also provide a way of comparing the graph with other population structures, such as the well-mixed population. In particular, and to the extent that our approximations are valid, graphs updated with a death-Birth process are more conducive to the evolution of cooperation than well-mixed populations for a large class of games (see condition (11)).

Second, we used numerical simulations to estimate the fixation probabilities and the difference in fixation probabilities of different strategies for particular examples of games (volunteer’s dilemma with and without cost sharing) and graphs (random regular graphs, rings, and lattices). Although simulations agree very well with the analytical approximations in the case of random regular graphs, discrepancies are evident in the case of rings and lattices, which are characterized by higher clustering and for which pair approximation is not sufficiently accurate. In these cases, the analytical approximations systematically overestimate the ability of a graph to promote the evolution of cooperation. Importantly, in the case of the volunteer’s dilemma without cost sharing and for rings or lattices of relatively large degree, the critical benefit-to-cost ratio above which cooperation is favored is greater, not smaller, than the corresponding value for a well-mixed population. Even though detrimental effects of spatial structure on cooperation have been previously noted in similar studies [62], our results are counterintuitive given the updating protocol and the intensity of selection we explored. Indeed, a death-Birth Moran process under weak selection would always favor cooperation (with respect to a well-mixed population of the same size) for any linear cooperation game, including any collection of two-player cooperation games. Our simulations show that this might not be the case when social dilemmas are instead modelled as nonlinear games such as the volunteer’s dilemma.

We used pair approximation and diffusion approximation to find approximate values for the structure coefficients, but other approaches can be used to obtain better estimates of them. In particular, coalescent theory [63] allows us to write the sigma rule in terms of selection coefficients (dependent on the payoffs of the game and the demographic parameters of the model) and expected coalescence times under neutrality [64, 65]; however, such expected coalescence times can be difficult to obtain exactly. Alternatively, for small graphs, the sigma rule and hence the structure coefficients can be explicitly calculated from the transition matrix of the evolutionary process (cf. Appendix C of Ref. [26]). Finally, we note that even in cases for which the structure coefficients are difficult to obtain by purely analytical means, they can be estimated numerically, either indirectly (by estimating the expected times to coalescence) or directly (by computing and comparing fixation probabilities).

For simplicity, we assumed that a focal player obtains its payoff from a single multiplayer game with its *k* immediate neighbors. Such assumption allowed us to consider multiplayer interactions on graphs in a straightforward way. However, this is in contrast with a common assumption of many studies of multiplayer spatial and network games in which a focal player’s total payoff is the sum of payoffs obtained in *k*+1 different games, one “centered” on the focal player itself and the other *k* centered on its neighbors [47–49]. As a result, focal players interact not only with first-order but also with second-order neighbors, which would lead to more intricate structure coefficients. For example, in this case the structure coefficients of a cycle are given by [51, 66]

$$\varsigma_{0}^{G^{*}}=\frac{N+1}{3(2N-3)},\;\varsigma_{1}^{G^{*}}=\frac{2N-1}{3(2N-3)},\;\varsigma_{2}^{G^{*}}=\frac{N-3}{2N-3}.$$ (22)

These values are different from those we calculated under the assumption that individuals play a single game with first-order neighbors, given by Eq (6). For *N* > 4, the structure coefficients fulfill $\varsigma^{G}\geq_{\text{con}}\varsigma^{G^{*}}$, meaning that our assumption of payoffs from a single game leads to less restrictive conditions for cooperation to be favored by selection. This observation is in line with previous results for pairwise games on graphs suggesting that the condition for the evolution of cooperation is optimized when interaction and replacement neighborhoods coincide [67], which corresponds to our assumption of individuals playing a single game. Future work should consider the calculation of structure coefficients for the cases where the payoff to a player also depends on games centered on neighbors and how the condition for the promotion of cooperation differs from the one resulting from our simplifying assumption.

We modelled social interactions as multiplayer matrix games with two discrete strategies (*A* and *B*) and obtained our results by assuming that selection is weak (*w* is small). Alternatively, one could model the same multiplayer game but assume instead that players can choose between two similar mixed strategies *z* and *z* + *δ*, where *z* and *z* + *δ* refer to the probability of playing *A* for each strategy, and *δ* is small [43, 68, 69]. In such a “*δ*-weak selection” scenario, and for any number of players, only a single structure coefficient is needed to identify conditions under which a higher probability of playing *A* is favored by natural selection. For transitive graphs of size *N* and degree *k*, this structure coefficient is given by [7, 25]

$$\sigma =\frac{(k+1)N-4k}{(k-1)N}.$$ (23)

Exchanging the structure coefficient *σ* for the “scaled relatedness coefficient” *κ* of inclusive fitness theory via the identity *κ* = (*σ* − 1)/(*σ*+1) [65], we obtain [16]

$$\kappa =\frac{N-2k}{k(N-2)}.$$ (24)

With such a value, recent results on multiplayer discrete games in structured populations under *δ*-weak selection [43] can be readily applied to show that, for all cooperation games as we defined them and for a death-Birth protocol, *A* is favored over *B* more easily for a graph-structured population than for a well-mixed population, as long as *N* > *k*+1. Such prediction qualitatively coincides with the one obtained from our analytical approximations, but does not capture our numerical results for the volunteer’s dilemma in rings and lattices.

To sum up, we have shown that even for multiplayer games on graphs, which are routinely analyzed by simulation only, some analytical insight can be generated. However, fully accounting for the complexity of evolutionary multiplayer games in graphs with high clustering remains a challenging open problem.

## Supporting Information

S1 Text Supplementary Methods. Calculations for the exact structure coefficients of cycles, the approximate structure coefficients of graphs of degree *k* ≥ 3, and a short description of the computational model used for our simulations.
(PDF)

S1 Fig Simulations of evolutionary game dynamics on graphs (difference in fixation probabilities, *N* = 500). Same as in Fig 3, but for a population size *N* = 500.
(PDF)

S2 FigSimulations of evolutionary game dynamics on graphs (fixation probabilities, *N* = 100).Open symbols show the fixation probability of cooperators (*ρ*_*A*_) and filled symbols the fixation probability of defectors (*ρ*_*B*_) as a function of the benefit-to-cost ratio B/C, for different types and degrees of the graph. Lines indicate analytical predictions for the fixation probabilities. Parameters: population size *N* = 100, intensity of selection *w* = 0.01, payoff cost C=1.(PDF)

S3 Fig Simulations of evolutionary game dynamics on graphs (fixation probabilities, *N* = 500). Same as in S2 Fig, but for a population size *N* = 500.
(PDF)
