# Supplementary material for: Evolutionary Games of Multiplayer Cooperation on Graphs
Source: PLoS Comput Biol. 2016 Aug 11;12(8):e1005059. doi: 10.1371/journal.pcbi.1005059 (PMC4981334; doi:10.1371/journal.pcbi.1005059)
Supplement: S1 Text — Calculations for the exact structure coefficients of cycles, the approximate structure coefficients of graphs of degree k ≥ 3, and a short description of the computational model used for our simulations. (PDF) [file pcbi.1005059.s001.pdf]

Supplementary Methods: Evolutionary games of multiplayer cooperation on graphs

Jorge Peña[1,‡,*], Bin Wu[2,1,‡], Jordi Arranz[1], Arne Traulsen[1]

1 Department of Evolutionary Theory, Max Planck Institute for Evolutionary Biology, Plön, Germany
2 School of Sciences, Beijing University of Posts and Telecommunications, Beijing, China

‡These authors contributed equally to this work.
*** pena@evolbio.mpg.de**

Contents

1 The cycle **2**

2 Regular graphs with $k \geq 3$ **3**

2.1 Pair approximation . . . 3
2.2 Updating a B-player . . . 4
2.3 Updating an A-player . . . 5
2.4 Separation of time scales . . . 6
2.5 Algebraic manipulations . . . 7
2.6 Diffusion approximation . . . 10
2.7 Fixation probabilities, sigma rule and structure coefficients . . . 12
2.8 Normalized structure coefficients . . . 14
2.9 A useful identity . . . 15

3 Computional model **16**

1 The cycle

We start by considering the cycle, for which $k = 2$, and where a single mutant always leads to a connected cluster of mutants. In this case, analytical expressions for the fixation probabilities of the two types and for the structure coefficients can be obtained exactly by adapting previous results on two-player games on cycles [1]. The state space of the stochastic process is $i = 0, \ldots, N$, where i is the number of A-players and N is the population size. At each time step, the number of i players either increases by one (with probability T_i^+), decreases by one (with probability T_i^-), or remains the same (with probability $1 - T_i^+ - T_i^-$). The fixation probability of a single mutant A is given by [2, 3]

$$\rho_A = \frac{1}{1 + \sum_{j=1}^{N-1} \prod_{i=1}^{j} \frac{T_i^-}{T_i^+}}, \tag{1}$$

and the ratio of the fixation probabilities is given by [2, 3]

$$\frac{\rho_A}{\rho_B} = \prod_{i=1}^{N-1} \frac{T_i^+}{T_i^-}. \tag{2}$$

In order to compute these quantities, we need to find expressions for the ratio of the transition probabilities, T_i^+/T_i^-, for each $i = 1, \ldots, N-1$. For convenience, let us define $\alpha_j = 1 - w + wa_j$ and $\beta_j = 1 - w + wb_j$ for $j = 0, 1, 2$, where w is the intensity of selection and a_j (b_j) is the payoff of an A-player (B-player) when playing against two other players, $j \in \{0, 1, 2\}$ of which are A-players. For a death-Birth protocol, we find

$$T_i^+ = \begin{cases} \frac{2}{N}\frac{\alpha_0}{\alpha_0+\beta_0} & \text{if } i = 1 \\ \frac{2}{N}\frac{\alpha_1}{\alpha_1+\beta_0} & \text{if } i = 2 \\ \frac{2}{N}\frac{\alpha_1}{\alpha_1+\beta_0} & \text{if } 3 \le i \le N-3 \\ \frac{2}{N}\frac{\alpha_1}{\alpha_1+\beta_1} & \text{if } i = N-2 \\ \frac{1}{N} & \text{if } i = N-1 \end{cases}$$

and

$$T_i^- = \begin{cases} \frac{1}{N} & \text{if } i = 1 \\ \frac{2}{N}\frac{\beta_1}{\alpha_1+\beta_1} & \text{if } i = 2 \\ \frac{2}{N}\frac{\beta_1}{\alpha_2+\beta_1} & \text{if } 3 \le i \le N-3 \\ \frac{2}{N}\frac{\beta_1}{\alpha_2+\beta_1} & \text{if } i = N-2 \\ \frac{2}{N}\frac{\beta_2}{\alpha_2+\beta_2} & \text{if } i = N-1 \end{cases},$$

so that the ratio of transition probabilities is given by

$$\frac{T_i^+}{T_i^-} = \begin{cases} \frac{2\alpha_0}{\alpha_0+\beta_0} & \text{if } i = 1 \\ \frac{\alpha_1(\alpha_1+\beta_1)}{\beta_1(\alpha_1+\beta_0)} & \text{if } i = 2 \\ \frac{\alpha_1(\alpha_2+\beta_1)}{\beta_1(\alpha_1+\beta_0)} & \text{if } 3 \le i \le N-3 \\ \frac{\alpha_1(\alpha_2+\beta_1)}{\beta_1(\alpha_1+\beta_1)} & \text{if } i = N-2 \\ \frac{\alpha_2+\beta_2}{2\beta_2} & \text{if } i = N-1 \end{cases}. \tag{3}$$

With the previous expression, and for weak selection ($w \ll 1$) we obtain

$$\begin{aligned}\rho_A \approx \frac{1}{N} + \frac{w}{4N^2} \{&2(N-1)a_0 + (N^2 - N - 4)a_1 + (N^2 - 5N + 6)a_2 \\ &-(N^2 - N - 6)b_0 - (N^2 - 3N + 4)b_1 - 2b_2\}.\end{aligned} \tag{4}$$

By symmetry, the expression for ρ_B can be obtained from the expression for ρ_A after replacing a_j by b_{k-j} and b_j by a_{k-j}, i.e.,

$$\rho_B \approx \frac{1}{N} + \frac{w}{4N^2} \left\{2(N-1)b_2 + (N^2 - N - 4)b_1 + (N^2 - 5N + 6)b_0 \right.$$
$$\left. -(N^2 - N - 6)a_2 - (N^2 - 3N + 4)a_1 - 2a_0 \right\}. \quad (5)$$

In a similar manner, for weak selection the ratio of fixation probabilities can be approximated by

$$\frac{\rho_A}{\rho_B} \approx 1 + \frac{w}{2} \{a_0 + (N-2)a_1 + (N-3)a_2 - (N-3)b_0 - (N-2)b_1 - b_2\}. \quad (6)$$

Thus, the condition $\rho_A > \rho_B$ becomes

$$\underbrace{1}_{\sigma_0}(a_0 - b_2) + \underbrace{(N-2)}_{\sigma_1}(a_1 - b_1) + \underbrace{(N-3)}_{\sigma_2}(a_2 - b_0) > 0,$$

from which we identify the structure coefficients:

$$\sigma_0 = 1,\ \sigma_1 = N-2,\ \sigma_3 = N-3.$$

As we assume $N \geq 3$, the structure coefficients are nonnegative. Normalizing the structure coefficients we obtain

$$\varsigma_0 = \frac{1}{2(N-2)},\ \varsigma_1 = \frac{1}{2},\ \varsigma_3 = \frac{N-3}{2(N-2)},$$

which are the values given by Eq. (6) in the main text.

2 Regular graphs with $k \geq 3$

For regular graphs with degree $k \geq 3$, we obtain the structure coefficients by finding an approximate expression for the comparison of fixation probabilities, $\rho_A > \rho_B$. To estimate these fixation probabilities, we follow closely the procedure used by Ohtsuki et al. [4], based on a combination of pair approximation and diffusion approximation.

2.1 Pair approximation

Let us denote by p_A and p_B the global frequencies of types A and B, by p_{AA}, p_{AB}, p_{BA} and p_{BB} the frequencies of AA, AB, BA, and BB pairs, and by $q_{X|Y}$ the conditional probability of finding an X-player given that the adjacent node is occupied by a Y-player, where X and Y stand for A or B. Such probabilities satisfy

$$p_A + p_B = 1, \quad (7a)$$
$$p_{AB} = p_{BA}, \quad (7b)$$
$$q_{A|X} + q_{B|X} = 1, \quad (7c)$$
$$q_{X|Y} = \frac{p_{XY}}{p_Y}, \quad (7d)$$

implying that the system can be described by only two variables: p_A and p_{AA}.

In probabilistic cellular automata such as the one analyzed here, the dynamics of frequencies of types (p_A, p_B) and pairs of types (p_{AA}, p_{AB}, p_{BA} and p_{BB}) will depend on triplets and higher-order spatial configurations [5]. Pair approximation allows us to obtain a closed system by approximating third- and higher-order spatial moments by heuristic expressions involving second- and first-order moments only [5, 6]. In particular, for each site X, we assume that the probability of finding j A-players among its k neighbors follows the binomial distribution

$$\binom{k}{j} q_{A|X}^{j} (1 - q_{B|X})^{k-j}. \tag{8}$$

Likewise, for each pair XY, we assume that the probability of finding j A-players among the $k-1$ neighbors of X not including Y follows the binomial distribution

$$\binom{k-1}{j} q_{A|X}^{j} (1 - q_{A|X})^{k-1-j}. \tag{9}$$

Here, we implicitly assume that members of a pair are unlikely to have common neighbors, as it is approximately the case for random graphs. In this case, Eq. (8) and Eq. (9) are standard (and parsimonious) assumptions (cf. Ref. [5], Eq. 19.27). For other graphs (such as lattices) the overlap among the neighbors of a pair introduce correlations not taken into account by our simplification.

In the following, we write down the change of p_A and p_{AA} under the assumptions of pair approximation. Then, we assume that selection is weak and that a separation of timescales holds in order to reduce the dimension of the system of equations. Finally, we employ a diffusion approximation to get the equation that governs the fixation probabilities. From the expressions of the fixation probabilities, the structure coefficients can be obtained after some cumbersome algebra.

2.2 Updating a B-player

A B-player is chosen to die with probability p_B; its k neighbors compete for the vacant vertex proportionally to their effective payoffs. Denoting by k_A and k_B the number of A and B players among these k neighbors, and by virtue of Eq. (8), the frequency of such configuration is given by

$$\binom{k}{k_A} {q_{A|B}}^{k_A} \left(1 - q_{A|B}\right)^{k-k_A}.$$

The effective payoff of each A-player connected by an edge to the dead B-player is given by

$$f_A^B = 1 - w + w\pi_A^B,$$

where

$$\pi_A^B = \sum_{j=0}^{k-1} \binom{k-1}{j} {q_{A|A}}^{j} \left(1 - q_{A|A}\right)^{k-1-j} a_j$$

is, by virtue of Eq. (9), the expected payoff to an A-player with one B co-player and $k-1$ other players.

Likewise, the effective payoff of each B-player connected by an edge to the dead B-player is given by

$$f_B^B = 1 - w + w\pi_B^B,$$

where

$$\pi_B^B = \sum_{j=0}^{k-1} \binom{k-1}{j} {q_{A|B}}^{j} \left(1 - q_{A|B}\right)^{k-1-j} b_j$$

is the expected payoff to a B-player with one B co-player and $k-1$ other players.

Under weak selection, the probability that a neighbor playing A replaces the vacant spot left by the dead B-player is given by

$$\frac{k_A f_A^B}{k_A f_A^B + k_B f_B^B} \approx \frac{k_A}{k} + w\frac{k_A(k-k_A)}{k^2}\mathcal{S}_B,$$

where

$$\begin{aligned}\mathcal{S}_B &= \pi_A^B - \pi_B^B \\ &= \sum_{j=0}^{k-1}\binom{k-1}{j} {q_{A|A}}^j \left(1-q_{A|A}\right)^{k-1-j} a_j - \sum_{j=0}^{k-1}\binom{k-1}{j} {q_{A|B}}^j \left(1-q_{A|B}\right)^{k-1-j} b_j. \end{aligned} \tag{10}$$

Hence, the frequency p_A of A-players in the population increases by $1/N$ with probability

$$\begin{aligned}\Pr\left(\Delta p_A = \frac{1}{N}\right) &= p_B \sum_{k_A=0}^{k}\binom{k}{k_A} {q_{A|B}}^{k_A}\left(1-q_{A|B}\right)^{k-k_A}\frac{k_A f_A^B}{k_A f_A^B + k_B f_B^B} \\ &\approx p_B\left\{q_{A|B} + w\frac{k-1}{k}q_{A|B}\left(1-q_{A|B}\right)\mathcal{S}_B\right\} \\ &= p_{AB}\left\{1 + w\frac{k-1}{k}q_{B|B}\mathcal{S}_B\right\}, \end{aligned} \tag{11}$$

where we used the formulas for the first two moments of a binomial distribution and the identities $p_B q_{A|B} = p_{AB}$ and $1 - q_{A|B} = q_{B|B}$ implied by Eq. (7).

Regarding pairs, if the B-player chosen to die is replaced by an A-player then the number of AA pairs increases by k_A. Since the total number of pairs in the population is equal to $kN/2$, the proportion p_{AA} of AA pairs increases by $2k_A/(kN)$ with probability

$$\Pr\left(\Delta p_{AA} = \frac{2k_A}{kN}\right) = p_B\binom{k}{k_A}{q_{A|B}}^{k_A}\left(1-q_{A|B}\right)^{k-k_A}\frac{k_A f_A^B}{k_A f_A^B + k_B f_B^B}.$$

2.3 Updating an A-player

An A-player is chosen to die with probability p_A. There are k_A A-players and k_B B-players in the neighborhood of the vacant node. The frequency of this configuration is (cf. Eq. (8))

$$\binom{k}{k_A}{q_{A|A}}^{k_A}\left(1-q_{A|A}\right)^{k-k_A}.$$

The effective payoff of each neighboring A-player is

$$f_A^A = 1 - w + w\pi_A^A,$$

where

$$\pi_A^A = \sum_{j=0}^{k-1}\binom{k-1}{j}{q_{A|A}}^j\left(1-q_{A|A}\right)^{k-1-j}a_{j+1}$$

is, by virtue of Eq. (9), the expected payoff to an A-player with one A co-player and $k-1$ other players.

Likewise, the effective payoff to each neighboring B-player is given by

$$f_B^A = 1 - w + w\pi_B^A,$$

where

$$\pi_B^A = \sum_{j=0}^{k-1} \binom{k-1}{j} {q_{A|B}}^j \left(1 - q_{A|B}\right)^{k-1-j} b_{j+1}$$

is the expected payoff to a B-player with one A co-player and $k-1$ other players.

The probability that one of the neighbors playing B replaces the vacancy is given by

$$\frac{k_B f_B^A}{k_A f_A^A + k_B f_B^A} \approx \frac{k_B}{k} + w\frac{k_B(k-k_B)}{k^2}\mathcal{S}_A,$$

where

$$\begin{aligned}\mathcal{S}_A &= \pi_B^A - \pi_A^A \\ &= \sum_{j=0}^{k-1} \binom{k-1}{j} {q_{A|B}}^j \left(1 - q_{A|B}\right)^{k-1-j} b_{j+1} - \sum_{j=0}^{k-1} \binom{k-1}{j} {q_{A|A}}^j \left(1 - q_{A|A}\right)^{k-1-j} a_{j+1}. \end{aligned} \tag{12}$$

The vacancy is replaced by a B-player and therefore p_A decreases by $1/N$ with probability

$$\begin{aligned}\Pr\left(\Delta p_A = -\frac{1}{N}\right) &= p_A \sum_{k_B=0}^{k} \binom{k}{k_B} {q_{A|A}}^{k-k_A} \left(1 - q_{A|A}\right)^{k_B} \frac{k_B f_B^A}{k_A f_A^A + k_B f_B^A} \\ &\approx p_A \left\{ q_{B|A} + w\frac{k-1}{k} q_{A|A}\left(1 - q_{A|A}\right)\mathcal{S}_A \right\} \\ &= p_{BA}\left\{1 + w\frac{k-1}{k} q_{A|A}\mathcal{S}_A\right\}. \end{aligned} \tag{13}$$

Regarding pairs, the proportion p_{AA} of AA pairs decreases by $2k_A/(kN)$ with probability

$$\Pr\left(\Delta p_{AA} = -\frac{2k_A}{kN}\right) = p_A \binom{k}{k_A} {q_{A|B}}^{k_A} \left(1 - q_{B|A}\right)^{k-k_A} \frac{k_B f_B^A}{k_A f_A^A + k_B f_B^A}.$$

2.4 Separation of time scales

Supposing that one replacement event takes place in one unit of time, the time derivative of p_A is given by

$$\dot{p}_A = \frac{1}{N}\Pr\left(\Delta p_A = \frac{1}{N}\right) - \frac{1}{N}\Pr\left(\Delta p_A = -\frac{1}{N}\right). \tag{14}$$

Using Eq. (11) and (13) we obtain, to first order in w:

$$\dot{p}_A = w\frac{k-1}{kN} p_{AB}\mathcal{S}$$

where

$$\mathcal{S} = q_{B|B}\mathcal{S}_B - q_{A|A}\mathcal{S}_A.$$

Similarly to Eq. (14), the time derivative of p_{AA} is given by

$$\begin{aligned}\dot{p}_{AA} &= \sum_{k_A=0}^{k}\left(\frac{2k_A}{kN}\right)\Pr\left(\Delta p_{AA}=\frac{2k_A}{kN}\right)+\sum_{k_A=0}^{k}\left(-\frac{2k_A}{kN}\right)\Pr\left(\Delta p_{AA}=-\frac{2k_A}{kN}\right)\\ &\approx \frac{2}{kN}p_{AB}\left[1+(k-1)\left(q_{A|B}-q_{A|A}\right)\right].\end{aligned}$$

For weak selection ($wk \ll 1$) the local density p_{AA} equilibrates much more quickly than the global density p_A. Therefore, the dynamical system rapidly converges onto the slow manifold where $\dot{p}_{AA}=0$ and hence

$$1+(k-1)\left(q_{A|B}-q_{A|A}\right)=0.$$

From this expression and Eq. (7) we obtain

$$q_{A|A}-q_{A|B}=q_{B|B}-q_{B|A}=r, \tag{15}$$

where we define

$$r=\frac{1}{k-1}. \tag{16}$$

As pointed out by Ohtsuki et al. [4], Eq. (15) measures the amount of positive correlation or effective assortment between adjacent players generated by the population structure. Moreover, expression (15) together with Eq. (7) leads to

$$q_{A|A}=p_A+r(1-p_A)=r+(1-r)p_A, \tag{17a}$$
$$q_{A|B}=(1-r)p_A, \tag{17b}$$
$$q_{B|A}=(1-r)(1-p_A), \tag{17c}$$
$$q_{B|B}=rp_A+(1-p_A)=r+(1-r)(1-p_A). \tag{17d}$$

2.5 Algebraic manipulations

It follows from the previous approximations that $\dot{p}_A$ is proportional to

$$\begin{aligned}\mathcal{S} &= q_{B|B}\mathcal{S}_B-q_{A|A}\mathcal{S}_A\\ &= \left[rp_A+(1-p_A)\right]\mathcal{S}_B-\left[p_A+r(1-p_A)\right]\mathcal{S}_A,\end{aligned} \tag{18}$$

which is a polynomial of degree k in p_A. Let us write such polynomial in a more compact form. To do so, we make use of the following identities:

$$\sum_{j=0}^{n}\binom{n}{j}\left[x+r(1-x)\right]^{j}\left[(1-r)(1-x)\right]^{n-j}a_j=\sum_{j=0}^{n}\binom{n}{j}x^j(1-x)^{n-j}\sum_{\ell=0}^{n-j}\binom{n-j}{\ell}r^{\ell}(1-r)^{n-j-\ell}a_{j+\ell} \tag{19}$$

$$\sum_{j=0}^{n}\binom{n}{j}\left[(1-r)x\right]^{j}\left[1-(1-r)x\right]^{n-j}a_j=\sum_{j=0}^{n}\binom{n}{j}x^j(1-x)^{n-j}\sum_{\ell=0}^{j}\binom{j}{\ell}r^{\ell}(1-r)^{j-\ell}a_{j-\ell} \tag{20}$$

$$x\sum_{j=0}^{n-1}\binom{n-1}{j}x^j(1-x)^{n-1-j}a_j=\sum_{j=0}^{n}\binom{n}{j}x^j(1-x)^{n-j}\frac{ja_{j-1}}{n}, \tag{21}$$

$$(1-x)\sum_{j=0}^{n-1}\binom{n-1}{j}x^j(1-x)^{n-1-j}a_j=\sum_{k=0}^{n}\binom{n}{j}x^j(1-x)^{n-j}\frac{(n-j)a_j}{n}. \tag{22}$$

Proofs of identities (21) and (22) are provided in Appendix B of Ref. [7]. In the following, we prove (19) [(20) is proven in a similar way]. Starting from the left side of (19) we expand the term $[x+r(1-x)]^j$ (by applying the binomial theorem) and rearrange to obtain:

$$\begin{aligned}\sum_{j=0}^{n}\binom{n}{j}\left[x+r(1-x)\right]^{j}\left[(1-r)(1-x)\right]^{n-j}a_j &= \sum_{j\geq 0}\binom{n}{j}\left[(1-r)(1-x)\right]^{n-j}a_j\sum_{\ell\geq 0}\binom{j}{\ell}\left[r(1-x)\right]^{\ell}x^{j-\ell}\\ &= \sum_{j\geq 0}\sum_{\ell\geq 0}\binom{n}{j}\binom{j}{\ell}x^{j-\ell}(1-x)^{n-(j-\ell)}r^{\ell}(1-r)^{n-j}a_j.\end{aligned}$$

Now, since

$$\binom{n}{j}\binom{j}{\ell}=\binom{n}{j}\binom{j}{j-\ell}=\binom{n}{j-\ell}\binom{n-(j-\ell)}{\ell}$$

and introducing $m=j-\ell$, we can write

$$\begin{aligned}\sum_{j\geq 0}\sum_{\ell\geq 0}\binom{n}{j}\binom{j}{\ell}x^{j-\ell}(1-x)^{n-(j-\ell)}r^{\ell}(1-r)^{n-j}a_j &= \sum_{j\geq 0}\sum_{m\geq 0}\binom{n}{m}\binom{n-m}{j-n}x^m(1-x)^{n-m}r^{j-m}(1-r)^{n-j}a_j\\ &= \sum_{m\geq 0}\binom{n}{m}x^m(1-x)^{n-m}\sum_{j\geq 0}\binom{n-m}{j-m}r^{j-m}(1-r)^{n-j}a_j.\end{aligned}$$

Replacing $\ell=j-m$ in the last sum,

$$\begin{aligned}&\sum_{m\geq 0}\binom{n}{m}x^m(1-x)^{n-m}\sum_{j\geq 0}\binom{n-m}{j-m}r^{j-m}(1-r)^{n-j}a_j\\ &=\sum_{m\geq 0}\binom{n}{m}x^m(1-x)^{n-m}\sum_{\ell\geq 0}\binom{n-m}{\ell}r^{\ell}(1-r)^{n-m-\ell}a_{m+\ell}.\end{aligned}$$

Finally, changing the dummy variable m by j in the last expression and making explicit the upper limits of the sums, we obtain the right side of (19).

Replacing Eq. (17a) and (17b) into Eq. (10), and applying identities (19) and (20), we can write

$$\begin{aligned}
\mathcal{S}_B(p_A) &= \sum_{j=0}^{k-1}\binom{k-1}{j} {q_{A|A}}^j \left(1-q_{A|A}\right)^{k-1-j} a_j - \sum_{j=0}^{k-1}\binom{k-1}{j} {q_{A|B}}^j \left(1-q_{A|B}\right)^{k-1-j} b_j \\
&= \sum_{j=0}^{k-1}\binom{k-1}{j} \left[p_A + r(1-p_A)\right]^j \left[(1-r)(1-p_A)\right]^{k-1-j} a_j \\
&- \sum_{j=0}^{k-1}\binom{k-1}{j} \left[(1-r)p_A\right]^j \left[1-(1-r)p_A\right]^{k-1-j} b_j \\
&= \sum_{j=0}^{k-1}\binom{k-1}{j} p_A^j (1-p_A)^{k-1-j} \sum_{\ell=0}^{k-1-j}\binom{k-1-j}{\ell} r^\ell (1-r)^{k-1-j-\ell} a_{j+\ell} \\
&- \sum_{j=0}^{k-1}\binom{k-1}{j} p_A^j (1-p_A)^{k-1-j} \sum_{\ell=0}^{j}\binom{j}{\ell} r^\ell (1-r)^{j-\ell} b_{j-\ell},
\end{aligned}$$

so that we obtain

$$\mathcal{S}_B(p_A) = \sum_{j=0}^{k-1}\binom{k-1}{j} p_A^j (1-p_A)^{k-1-j} c_j, \tag{23}$$

where

$$c_j = \sum_{\ell=0}^{k-1-j}\binom{k-1-j}{\ell} r^\ell (1-r)^{k-1-j-\ell} a_{j+\ell} - \sum_{\ell=0}^{j}\binom{j}{\ell} r^\ell (1-r)^{j-\ell} b_{j-\ell}. \tag{24}$$

Likewise, replacing Eq. (17) into Eq. (12), and applying identities (19) and (20) we obtain

$$\begin{aligned}
\mathcal{S}_A(p_A) &= \sum_{j=0}^{k-1}\binom{k-1}{j} {q_{A|B}}^j \left(1-q_{A|B}\right)^{k-1-j} b_{j+1} - \sum_{j=0}^{k-1}\binom{k-1}{j} q_{A|A}^j \left(1-q_{A|A}\right)^{k-1-j} a_{j+1} \\
&= \sum_{j=0}^{k-1}\binom{k-1}{j} \left[(1-r)p_A\right]^j \left[1-(1-r)p_A\right]^{k-1-j} b_{j+1} \\
&- \sum_{j=0}^{k-1}\binom{k-1}{j} \left[p_A + r(1-p_A)\right]^j \left[(1-r)(1-p_A)\right]^{k-1-j} a_{j+1} \\
&= \sum_{j=0}^{k-1}\binom{k-1}{j} p_A^j (1-p_A)^{k-1-j} \sum_{\ell=0}^{j}\binom{j}{\ell} r^\ell (1-r)^{j-\ell} b_{j+1-\ell} \\
&- \sum_{j=0}^{k-1}\binom{k-1}{j} p_A^j (1-p_A)^{k-1-j} \sum_{\ell=0}^{k-1-j}\binom{k-1-j}{\ell} r^\ell (1-r)^{k-1-j-\ell} a_{j+1+\ell},
\end{aligned}$$

and hence

$$\mathcal{S}_A(p_A) = -\sum_{j=0}^{k-1} \binom{k-1}{j} p_A^j (1-p_A)^{k-1-j} d_j, \tag{25}$$

where

$$d_j = \sum_{\ell=0}^{k-1-j} \binom{k-1-j}{\ell} r^\ell (1-r)^{k-1-j-\ell} a_{j+1+\ell} - \sum_{\ell=0}^{j} \binom{j}{\ell} r^\ell (1-r)^{j-\ell} b_{j+1-\ell}. \tag{26}$$

Replacing Eq. (23) and Eq. (25) into Eq. (18), and applying identities (21) and (22), we finally obtain

$$\begin{aligned}
\mathcal{S}(p_A) &= [rp_A + (1-p_A)]\,\mathcal{S}_B - [p_A + r(1-p_A)]\,\mathcal{S}_A \\
&= rp_A \sum_{j=0}^{k-1} \binom{k-1}{j} p_A^j (1-p_A)^{k-1-j} c_j + (1-p_A) \sum_{j=0}^{k-1} \binom{k-1}{j} p_A^j (1-p_A)^{k-1-j} c_j \\
&\quad + p_A \sum_{j=0}^{k-1} \binom{k-1}{j} p_A^j (1-p_A)^{k-1-j} d_j + r(1-p_A) \sum_{j=0}^{k-1} \binom{k-1}{j} p_A^j (1-p_A)^{k-1-j} d_j \\
&= r \sum_{j=0}^{k} \binom{k}{j} p_A^j (1-p_A)^{k-j} \frac{jc_{j-1}}{k} + \sum_{j=0}^{k} \binom{k}{j} p_A^j (1-p_A)^{k-j} \frac{(k-j)c_j}{k} \\
&\quad + \sum_{j=0}^{k} \binom{k}{j} p_A^j (1-p_A)^{k-j} \frac{jd_{j-1}}{k} + r \sum_{j=0}^{k} \binom{k}{j} p_A^j (1-p_A)^{k-j} \frac{(k-j)d_j}{k},
\end{aligned}$$

and hence

$$\mathcal{S}(p_A) = \sum_{j=0}^{k} \binom{k}{j} p_A^j (1-p_A)^{k-j} e_j, \tag{27}$$

where

$$e_j = \frac{rjc_{j-1} + (k-j)c_j + jd_{j-1} + r(k-j)d_j}{k}. \tag{28}$$

2.6 Diffusion approximation

Assuming that Eq. (17) holds, we study a one dimensional diffusion process on the variable p_A. Therefore, within a short interval, Δt, we have

$$\begin{aligned}
\mathrm{E}[\Delta p_A] &\approx w \frac{k-2}{kN} p_A(1-p_A)\mathcal{S}(p_A)\Delta t \left(\equiv m(p_A)\Delta t\right), \\
\mathrm{Var}[\Delta p_A] &\approx \frac{2}{N^2} \frac{k-2}{k-1} p_A(1-p_A)\Delta t \left(\equiv v(p_A)\Delta t\right).
\end{aligned}$$

The fixation probability, $\phi_A(y)$ of strategy A with initial frequency $p_A(t=0) = y$, is then governed by the differential equation (cf. Eq. 4.13 in Ref. [8])

$$m(y) \frac{d\phi_A(y)}{dy} + \frac{v(y)}{2} \frac{d^2\phi_A(y)}{dy^2} = 0$$

with boundary conditions $\phi_A(0) = 0$ and $\phi_A(1) = 1$.

The probability that absorption eventually occurs at $p_A = 1$ is then (cf. Eq. 4.17 in Ref. [8])

$$\phi_A(y) = \frac{\int_0^y \psi(x)dx}{\int_0^1 \psi(x)dx},$$

where (cf. Eq. 4.16 in Ref. [8])

$$\psi(x) = \exp\left(-\int^x 2\frac{m(z)}{v(z)}dz\right) = \exp\left(-\frac{wN(k-1)}{k}\int^x \mathcal{S}(z)dz\right).$$

Since we assume that w is very small,

$$\phi_A(y) \approx y + \frac{wN(k-1)}{k}\left(y\int_0^1\int_0^x \mathcal{S}(z)dzdx - \int_0^y\int_0^x \mathcal{S}(z)dzdx\right).$$

This expression involves integrals of $\mathcal{S}(z)$. Using the formula for the integral of a polynomial in Bernstein form (cf. p. 391 of Ref. [9]), i.e.,

$$\int_0^x \sum_{j=0}^{n}\binom{n}{j} z^j(1-z)^{n-j}a_j\mathrm{d}z = \frac{1}{n+1}\sum_{j=0}^{n+1}\binom{n+1}{j}x^j(1-x)^{n+1-j}\sum_{\ell=0}^{j-1}a_\ell,$$

we obtain

$$\begin{aligned}\int_0^y\int_0^x \mathcal{S}(z)\mathrm{d}z\mathrm{d}x &= \int_0^y\int_0^x\sum_{j=0}^{k}\binom{k}{j}z^j(1-z)^{k-j}e_j\mathrm{d}z\mathrm{d}x\\ &= \int_0^y \frac{1}{k+1}\sum_{j=0}^{k+1}\binom{k+1}{j}x^j(1-x)^{k+1-j}\sum_{\ell=0}^{j-1}e_\ell\mathrm{d}x\\ &= \frac{1}{k+1}\frac{1}{k+2}\sum_{j=0}^{k+2}\binom{k+2}{j}y^j(1-y)^{k+2-j}\sum_{m=0}^{j-1}\sum_{\ell=0}^{m-1}e_\ell \qquad (29)\end{aligned}$$

and hence

$$\begin{aligned}\int_0^1\int_0^x \mathcal{S}(z)\mathrm{d}z\mathrm{d}x &= \frac{1}{(k+2)(k+1)}\sum_{m=0}^{k+1}\sum_{\ell=0}^{m-1}e_\ell\\ &= \frac{1}{(k+2)(k+1)}\sum_{m=0}^{k}\sum_{\ell=0}^{m}e_\ell\\ &= \frac{1}{(k+2)(k+1)}\sum_{j=0}^{k}(k+1-j)e_j.\end{aligned}$$

Writing out Eq. (29) as

$$\begin{aligned}&\frac{1}{(k+2)(k+1)}\left[0+0+\binom{k+2}{2}y^2(1-y)^k\left(\sum_{m=0}^{1}\sum_{\ell=0}^{m-1}e_\ell\right)+\dots\right]\\ &= \frac{1}{(k+2)(k+1)}\frac{(k+2)!}{k!2!}y^2(1-y)^k e_0+\dots\\ &= \frac{1}{2}y^2(1-y)^k e_0+\dots\end{aligned}$$

it is clear that, for $y = 1/N$ and N large, $\int_0^y \int_0^x \mathcal{S}(z)\mathrm{d}z\mathrm{d}x$ can be approximated by

$$\frac{e_0}{2N^2}.$$

The fixation probability, $\rho_A = \phi_A(1/N)$, can then be written as

$$\begin{aligned}
\rho_A &\approx \frac{1}{N} + \frac{wN(k-1)}{k}\left(\frac{1}{N}\frac{1}{(k+2)(k+1)}\sum_{j=0}^{k}(k+1-j)e_j - \frac{e_0}{2N^2}\right)\\
&= \frac{1}{N} + \frac{w(k-1)}{(k+2)(k+1)k}\left(\sum_{j=0}^{k}(k+1-j)e_j - (k+2)(k+1)\frac{e_0}{2N}\right)\\
&= \frac{1}{N} + \frac{w(k-1)}{(k+2)(k+1)k}\left(\sum_{j=1}^{k}(k+1-j)e_j + (k+1)e_0\left(1-\frac{k+2}{2N}\right)\right)
\end{aligned}$$

If $k \ll N$, then $(k+2)/(2N) \ll 1$, and we finally obtain

$$\rho_A \approx \frac{1}{N} + w\frac{k-1}{(k+2)(k+1)k}\sum_{j=0}^{k}(k+1-j)e_j. \tag{30}$$

2.7 Fixation probabilities, sigma rule and structure coefficients

From Eq. (30), the fixation probability of a mutant A is greater than neutral if $\sum_{j=0}^{k}(k+1-j)e_j > 0$. By Eq. (28), the coefficients e_j are linear in c_j and d_j, which are linear in the payoff entries a_j and b_j (cf. Eq. (24) and (26)). Thus, $\sum_{j=0}^{k}(k+1-j)e_j$ is linear in the payoff entries, meaning that there exist α_j and β_j such that

$$\sum_{j=0}^{k}(k+1-j)e_j = \sum_{j=0}^{k}(\alpha_j a_j + \beta_j b_j),$$

and so

$$\rho_A \approx \frac{1}{N} + w\frac{k-1}{(k+2)(k+1)k}\sum_{j=0}^{k}(\alpha_j a_j + \beta_j b_j). \tag{31}$$

By symmetry, the fixation probability of a single B mutant is given by

$$\rho_B \approx \frac{1}{N} + w\frac{k-1}{(k+2)(k+1)k}\sum_{j=0}^{k}\left(\alpha_j b_{k-j} + \beta_j a_{k-j}\right). \tag{32}$$

Therefore, under weak selection

$$\rho_A > \rho_B \Leftrightarrow \sum_{j=0}^{k}\left(\alpha_j a_j + \beta_j b_j\right) > \sum_{j=0}^{k}\left(\alpha_j b_{k-j} + \beta_j a_{k-j}\right) \Leftrightarrow \sum_{j=0}^{k}\sigma_j\left(a_j - b_{k-j}\right) > 0, \tag{33}$$

where

$$\sigma_j = \alpha_j - \beta_{k-j}. \tag{34}$$

The rightmost expression in Eq. (33) has been termed the "sigma rule" and the coefficients σ_j are the structure coefficients [10–12].

To obtain expressions for the fixation probabilities and the structure coefficients, we need to calculate α_j and β_j. For a multiplayer game with $a_j = \delta_{i,j}$ (i.e., $a_j = 1$ for some $j = i$ and $a_j = 0$ otherwise), we have

$$\alpha_i = \sum_{j=0}^{k} (k+1-j) e_j^i, \tag{35}$$

where e_j^i denotes the coefficient e_j with $a_j = \delta_{i,j}$ and $b_j = 0$ for all j. Replacing the formula for e_j (28) into Eq. (35), expressing r in terms of k (Eq. (16)), and simplifying we get

$$\begin{aligned}
\alpha_i &= \frac{r}{k}\sum_{j=0}^{k}(k+1-j)jc_{j-1}^i + \frac{1}{k}\sum_{j=0}^{k}(k+1-j)(k-j)c_j^i + \frac{1}{k}\sum_{j=0}^{k}(k+1-j)jd_{j-1}^i + \frac{r}{k}\sum_{j=0}^{k}(k+1-j)(k-j)d_j^i \\
&= \frac{r}{k}\sum_{j=0}^{k}(k-j)(j+1)c_j^i + \frac{1}{k}\sum_{j=0}^{k}(k+1-j)(k-j)c_j^i + \frac{1}{k}\sum_{j=0}^{k}(k-j)(j+1)d_j^i + \frac{r}{k}\sum_{j=0}^{k}(k+1-j)(k-j)d_j^i \\
&= \frac{1}{k}\sum_{j=0}^{k-1}(k-j)\left[r(j+1)+(k+1-j)\right]c_j^i + \frac{1}{k}\sum_{j=0}^{k-1}(k-j)\left[(j+1)+r(k+1-j)\right]d_j^i \\
&= \frac{1}{k(k-1)}\sum_{j=0}^{k-1}(k-j)\left\{\left[k^2-(k-2)j\right]c_j^i + \left[2k+(k-2)j\right]d_j^i\right\}.
\end{aligned}$$

Now, since for α_i we have that $a_j = \delta_{i,j}$ and $b_j = 0$ for all j, and from Eq. (24), (26), and Eq. (16), we have

$$\begin{aligned}
c_j^i &= \binom{k-1-j}{i-j}\left(\frac{1}{k-1}\right)^{i-j}\left(\frac{k-2}{k-1}\right)^{k-1-i} = \binom{k-1-j}{i-j}\frac{(k-2)^{k-1-i}}{(k-1)^{k-1-j}} \\
d_j^i &= \binom{k-1-j}{i-j-1}\left(\frac{1}{k-1}\right)^{i-j-1}\left(\frac{k-2}{k-1}\right)^{k-i} = \binom{k-1-j}{i-j-1}\frac{(k-2)^{k-i}}{(k-1)^{k-1-j}},
\end{aligned}$$

and $d_j^0 = 0$ for all $0 \le j \le k-1$. Hence

$$\begin{aligned}
\alpha_i = \frac{1}{k(k-1)}\sum_{j=0}^{k-1}(k-j)&\left\{\left[k^2-(k-2)j\right]\binom{k-1-j}{i-j}\frac{(k-2)^{k-1-i}}{(k-1)^{k-1-j}}\right. \\
&\left.+\left[2k+(k-2)j\right]\binom{k-1-j}{i-j-1}\frac{(k-2)^{k-i}}{(k-1)^{k-1-j}}\right\}.
\end{aligned} \tag{36}$$

Similarly (now letting the payoff entries be $b_j = \delta_{j,i}$ and $a_j = 0$) we obtain

$$\beta_i = \frac{1}{k(k-1)} \sum_{j=0}^{k-1} (k-j) \left\{ \left[k^2 - (k-2)j\right] (-1) \binom{j}{j-i} r^{j-i} (1-r)^i \right.$$
$$\left. + \left[2k + (k-2)j\right] (-1) \binom{j}{j-i+1} r^{j-i+1} (1-r)^{i-1} \right\}$$
$$= -\frac{1}{k(k-1)} \sum_{j=0}^{k-1} (k-j) \left\{ \left[k^2 - (k-2)j\right] \binom{j}{j-i} \frac{(k-2)^i}{(k-1)^j} \right.$$
$$\left. + \left[2k + (k-2)j\right] \binom{j}{j-i+1} \frac{(k-2)^{i-1}}{(k-1)^j} \right\}. \tag{37}$$

Replacing expressions (36) and (37) into Eq. (34) and simplifying, we finally obtain the following expressions for the structure coefficients

$$\sigma_j = \frac{(k-2)^{k-1-j}}{k(k-1)} \sum_{\ell=0}^{k-1} (k-\ell) \left\{ \left[k^2 - (k-2)\ell\right] \upsilon_{\ell,j,k} + \left[2k + (k-2)\ell\right] \tau_{\ell,j,k} \right\}, \tag{38}$$

where

$$\upsilon_{\ell,j,k} = \binom{k-1-\ell}{k-1-j} \frac{1}{(k-1)^{k-1-\ell}} + \binom{\ell}{k-j} \frac{k-2}{(k-1)^\ell}, \tag{39}$$

$$\tau_{\ell,j,k} = \binom{k-1-\ell}{k-j} \frac{k-2}{(k-1)^{k-1-\ell}} + \binom{\ell}{k-1-j} \frac{1}{(k-1)^\ell}. \tag{40}$$

2.8 Normalized structure coefficients

The structure coefficients given by Eq. (38) are nonnegative. Once we have an expression for their sum, we can normalize the structure coefficients so that they describe a probability distribution. In the following we work out such an expression.

We start by noting that, subtracting Eq. (32) from Eq. (31), the difference of the fixation probabilities under our approximations can be written as

$$\rho_A - \rho_B \approx w \frac{k-1}{(k+2)(k+1)k} \sum_{j=0}^{k} \sigma_j (a_j - b_{k-j}). \tag{41}$$

In particular, this expression holds for a multiplayer game with payoffs given by $a_j = 1$ and $b_j = 0$ for all j, for which Eq. (41) reduces to

$$\rho_A - \rho_B \approx w \frac{k-1}{(k+2)(k+1)k} \sum_{j=0}^{k} \sigma_j. \tag{42}$$

The multiplayer game with payoffs $a_j = 1$ and $b_j = 0$ for all j is mathematically equivalent to a collection of pairwise games played with neighbors with a payoff matrix

$$\begin{array}{cc} & \begin{array}{cc} A & B \end{array} \\ \begin{array}{c} A \\ B \end{array} & \begin{pmatrix} a & b \\ c & d \end{pmatrix}, \end{array}$$

where $a = b = 1/k$ and $c = d = 0$. Indeed, for such payoff values the accumulated payoff to an A-player is always 1 and that of a B-player is always 0. For a general pairwise game, we have that (cf. Eqs. (19) and (21) in the Supplementary Material of Ref. [4])

$$\rho_A \approx \frac{1}{N} + \frac{w}{6k}\left[(k^2+2k+1)a + (2k^2-2k-1)b - (k^2-k+1)c - (2k^2+k-1)d\right]. \quad (43)$$

By symmetry:

$$\rho_B \approx \frac{1}{N} + \frac{w}{6k}\left[(k^2+2k+1)d + (2k^2-2k-1)c - (k^2-k+1)b - (2k^2+k-1)a\right]. \quad (44)$$

Therefore, for $a = b = 1/k$ and $c = d = 0$, we have that

$$\rho_A - \rho_B \approx w. \quad (45)$$

Since the right hand side of Eq. (42) should be equal to the right hand side of Eq. (45), we conclude that

$$\sum_{j=0}^{k} \sigma_j = \frac{(k+2)(k+1)k}{k-1}.$$

Defining

$$\varsigma_i = \frac{\sigma_i}{\sum_{j=0}^{k} \sigma_j} = \frac{k-1}{(k+2)(k+1)k}\sigma_i, \quad (46)$$

we finally obtain

$$\varsigma_j = \frac{(k-2)^{k-1-j}}{(k+2)(k+1)k^2} \sum_{\ell=0}^{k-1} (k-\ell)\left\{\left[k^2-(k-2)\ell\right] \upsilon_{\ell,j,k} + \left[2k+(k-2)\ell\right]\tau_{\ell,j,k}\right\},$$

which is the expression for the normalized structure coefficients as given in Eq. (8) of the main text.

2.9 A useful identity

If individuals play the pairwise game

$$\begin{array}{cc} & \begin{array}{cc} A & B \end{array} \\ \begin{array}{c} A \\ B \end{array} & \begin{pmatrix} 1 & 0 \\ 0 & 0 \end{pmatrix} \end{array}$$

with each neighbor, then by Eqs. (43) and (44) we have

$$\rho_A - \rho_B \approx w\frac{k+1}{2}. \quad (47)$$

Now consider the difference in fixation probabilities arising from the equivalent multiplayer version, for which $a_j = j$ and $b_j = 0$ for all j. Replacing $a_j = j$ and $b_j = 0$ into Eq. (41) leads to

$$\rho_A - \rho_B \approx w\frac{k-1}{(k+2)(k+1)k} \sum_{i=0}^{k} \sigma_j j,$$

which by Eq. (46) can be written as

$$\rho_A - \rho_B \approx w \sum_{j=0}^{k} \varsigma_j j. \tag{48}$$

in terms of the normalized structure coefficients. Comparing Eq. (47) and (48), we finally obtain

$$\sum_{j=0}^{k} \varsigma_j j = \frac{k+1}{2},$$

which is the expression in Eq. (14) in the main text. Note that this expression is valid only in the limit of large N.

3 Computional model

We implemented numerical simulations of a Moran process with death-Birth (dB) updates for different kinds of graphs. The simulations rely on three different types of graphs: random regular, ring and lattice. We employ the C version of the igraph library[1] to generate all random regular graphs —igraph_k_regular_game()—, the ring of degree $k = 2$ —igraph_ring()— and the lattice of degree $k = 4$ —igraph_lattice(). Given that igraph does not provide generators for lattices of $k > 4$, we implemented an algortihm that extends a lattice of degree $k = 4$ (von Neumann neighborhood) to degrees $k = 6$ (hexagonal lattice) and $k = 8$ (Moore neighborhood). Similarly, we extend the ring of degree $k = 2$ by increasing its connectivity accordingly to generate cycles of degrees $k = 4, 6, 8, 10$.

At each realization of the simulation we start with a monomorphic population playing one of the two strategies and add a single mutant of the opposite strategy in a randomly chosen vertex. We allow the simulation to run until it reaches an absorbing state (i.e., when either of the two strategies reaches fixation). At each simulation step a vertex (*a*) is randomly selected from the whole population (i.e., death step) and a second vertex (*b*) is selected from the neighborhood of *a* with a probability proportional to its fitness. During this step we use the stochastic acceptance algorithm [13] to select an individual with a probability proportional to its fitness. Hereafter, the strategy of vertex *b* is copied to *a* (i.e., death step). The payoffs of the nodes —which depend on the game in place, their own strategies, and the strategies of their neighbours— are calculated as discussed in the main text. For optimization purposes, in the first step of each realisation we compute the payoffs of the whole network. Thenceforth we only re-compute the payoff of a vertex and its neighbors whenever a vertex switches its strategy.

We repeat this process for 10^7 different realizations and keep track of the number of times the mutant strategy has reached fixation. At the final step we compute the fixation probability of the mutant strategy as the ratio between the number of hits —i.e., number of times the mutant invaded the resident strategy— and the total number of realizations. We run separate simulation batches for both strategies, in a way that both strategies play as the mutant and resident.

References

1. Ohtsuki H, Nowak MA. Evolutionary games on cycles. Proceedings of the Royal Society B. 2006;273:2249–2256.

[1] http://igraph.org/

2. Karlin S, Taylor HMA. A First Course in Stochastic Processes. 2nd ed. London: Academic; 1975.
3. Traulsen A, Hauert C. Stochastic evolutionary game dynamics. In: Schuster HG, editor. Reviews of Nonlinear Dynamics and Complexity. vol. II. Weinheim: Wiley-VCH; 2009. p. 25–61.
4. Ohtsuki H, Hauert C, Lieberman E, Nowak MA. A simple rule for the evolution of cooperation on graphs. Nature. 2006;441:502–505.
5. van Baalen M. Pair approximations for different spatial geometries. In: The geometry of ecological interactions: simplifying spatial complexity. vol. 742. Cambridge University Press Cambridge; 2000. p. 359–387.
6. Matsuda H, Ogita N, Sasaki A, Sato K. Statistical Mechanics of Populations. Progress of Theoretical Physics. 1992;88(6):1035–1049.
7. Peña J, Nöldeke G, Lehmann L. Evolutionary dynamics of collective action in spatially structured populations. Journal of Theoretical Biology. 2015;382:122–136.
8. Ewens WJ. Mathematical Population Genetics. I. Theoretical Introduction. New York: Springer; 2004.
9. Farouki RT. The Bernstein polynomial basis: A centennial retrospective. Computer Aided Geometric Design. 2012;29:379–419.
10. Tarnita CE, Ohtsuki H, Antal T, Fu F, Nowak MA. Strategy selection in structured populations. Journal of Theoretical Biology. 2009;259:570–581.
11. Wu B, Traulsen A, Gokhale CS. Dynamic properties of evolutionary multi-player games in finite populations. Games. 2013;4(2):182–199.
12. Peña J, Wu B, Traulsen A. Ordering structured populations in multiplayer cooperation games. Journal of the Royal Society Interface. 2016;13:20150881.
13. Lipowski A, Lipowska D. Roulette-wheel selection via stochastic acceptance. Physica A: Statistical Mechanics and its Applications. 2012 March;391(6):2193–2196.
