# Supplementary material for: Evolutionary Games of Multiplayer Cooperation on Graphs
Source: PLoS Comput Biol. 2016 Aug 11;12(8):e1005059. doi: 10.1371/journal.pcbi.1005059 (PMC4981334; doi:10.1371/journal.pcbi.1005059)
Supplement: S1 Fig — Same as in Fig 3, but for a population size N = 500. (PDF) [file pcbi.1005059.s002.pdf]

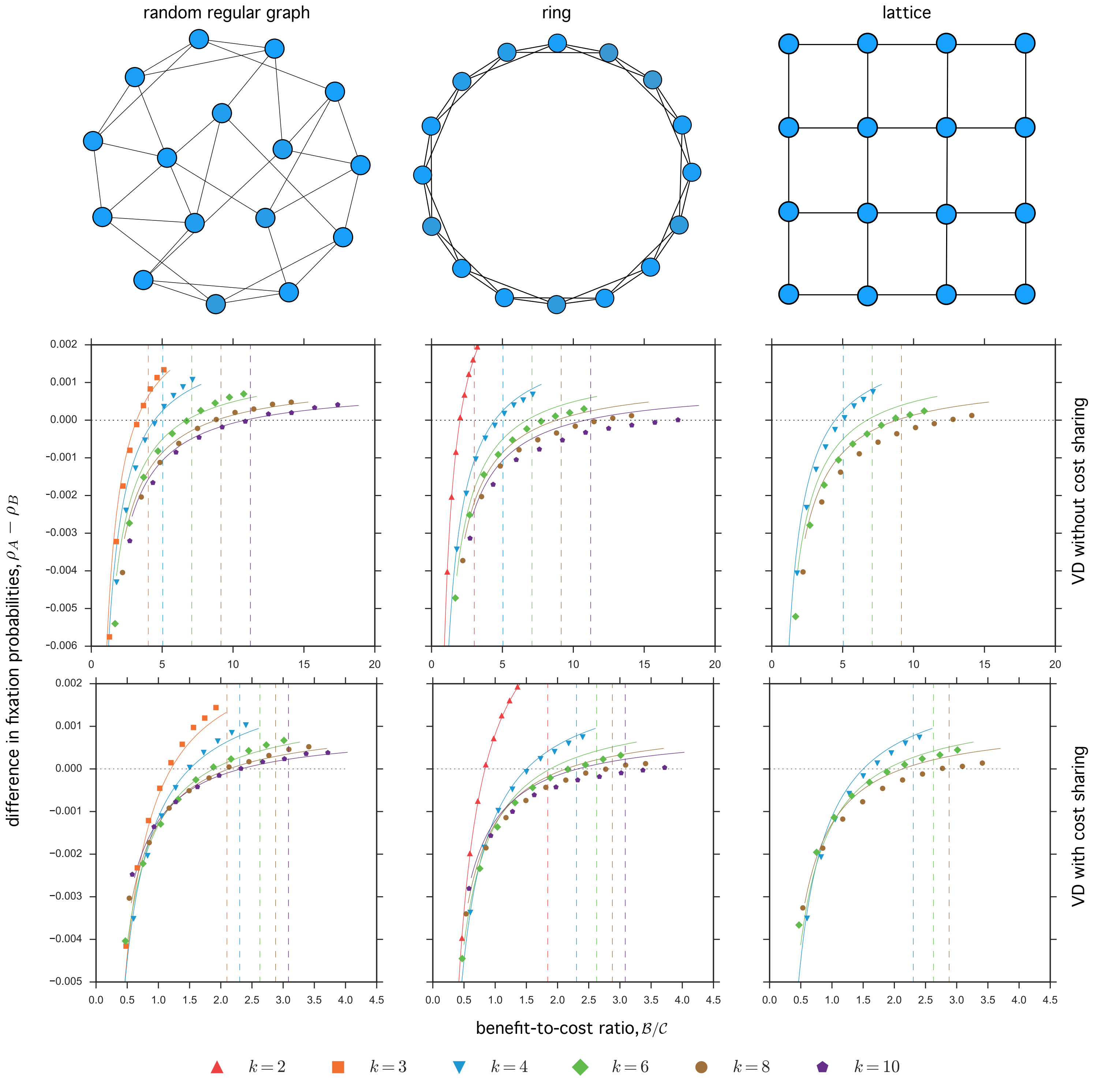

random regular graph
ring
lattice
VD without cost sharing
VD with cost sharing
difference in fixation probabilities, $\rho_A - \rho_B$
benefit-to-cost ratio, $\mathcal{B}/\mathcal{C}$
$k = 2$
$k = 3$
$k = 4$
$k = 6$
$k = 8$
$k = 10$
