# Supplementary figures and images for: Evolutionary Games of Multiplayer Cooperation on Graphs
Source: PLoS Comput Biol. 2016 Aug 11;12(8):e1005059. doi: 10.1371/journal.pcbi.1005059 (PMC4981334; doi:10.1371/journal.pcbi.1005059)

random regular graph

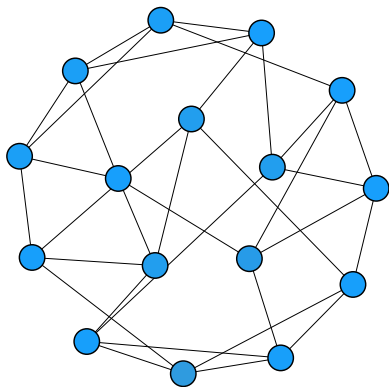

ring

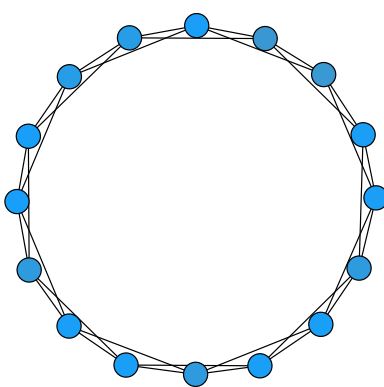

lattice

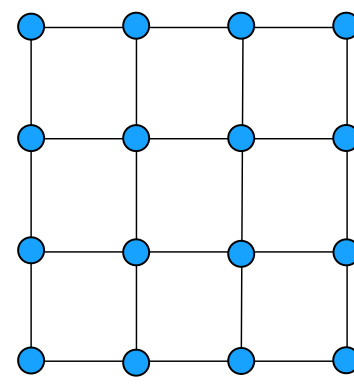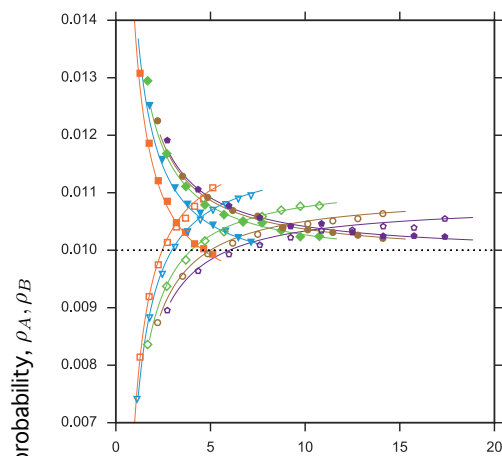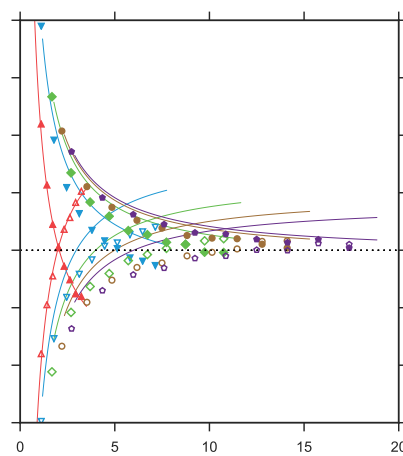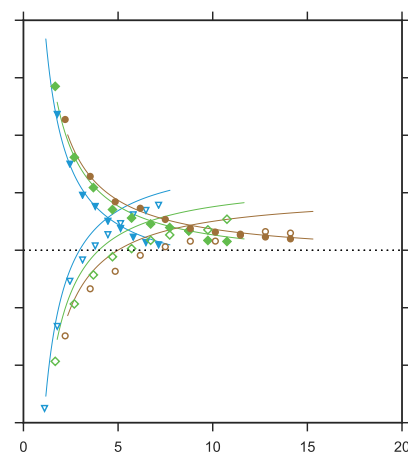

VD without cost sharing

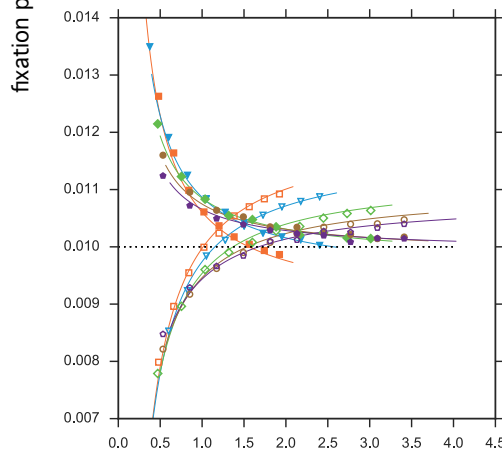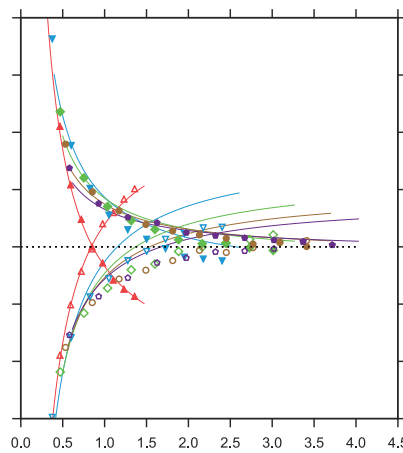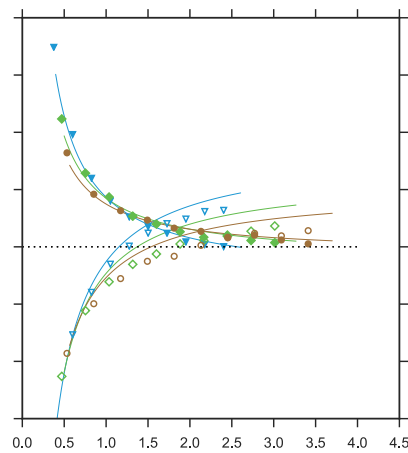

VD with cost sharing

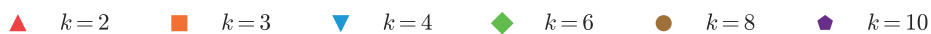

Supplement: S2 Fig — Open symbols show the fixation probability of cooperators (ρA) and filled symbols the fixation probability of defectors (ρB) as a function of the benefit-to-cost ratio B/C, for different types and degrees of the graph. Lines indicate analytical predictions for the fixation probabilities. Parameters: population size N = 100, intensity of selection w = 0.01, payoff cost C=1. (PDF) [file pcbi.1005059.s003.pdf]
