# Supplementary material for: Evolutionary Games of Multiplayer Cooperation on Graphs
Source: PLoS Comput Biol. 2016 Aug 11;12(8):e1005059. doi: 10.1371/journal.pcbi.1005059 (PMC4981334; doi:10.1371/journal.pcbi.1005059)
Supplement: S3 Fig — Same as in S2 Fig, but for a population size N = 500. (PDF) [file pcbi.1005059.s004.pdf]

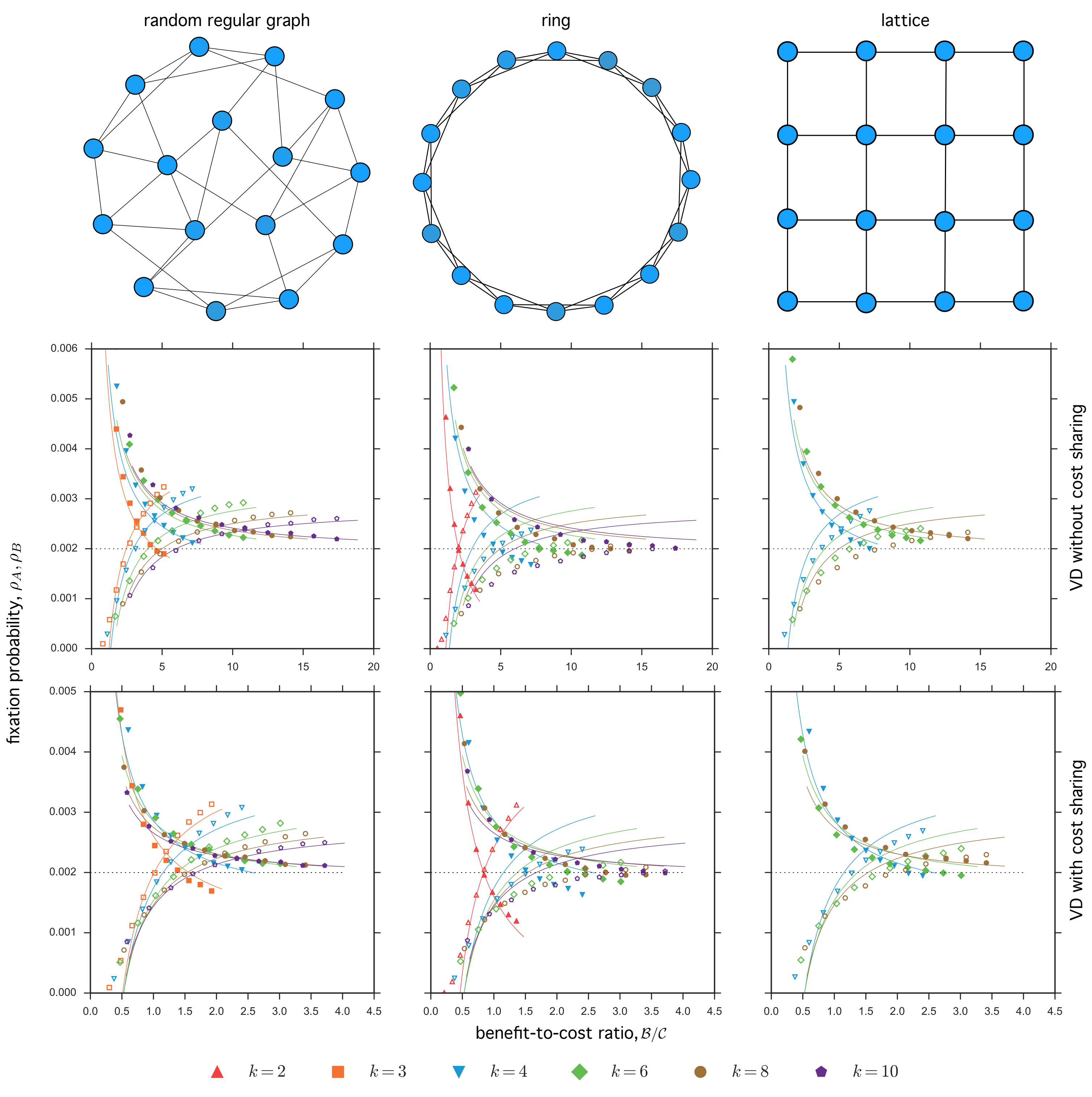

random regular graph
ring
lattice
fixation probability, ρ_A, ρ_B
VD without cost sharing
VD with cost sharing
benefit-to-cost ratio, $\mathcal{B}/\mathcal{C}$
$k=2$
$k=3$
$k=4$
$k=6$
$k=8$
$k=10$
